# Mouse Gamma Herpesvirus MHV-68 Induces Severe Gastrointestinal (GI) Dilatation in Interferon Gamma Receptor-Deficient Mice (IFNγR^−/−^) That Is Blocked by Interleukin-10

**DOI:** 10.3390/v10100518

**Published:** 2018-09-23

**Authors:** Hao Chen, Mee Yong Bartee, Jordan R. Yaron, Liying Liu, Liqiang Zhang, Donghang Zheng, Ian B. Hogue, Whitney L. Bullard, Scott Tibbetts, Alexandra R. Lucas

**Affiliations:** 1 Department of Medicine, Divisions of Cardiovascular Medicine and Rheumatology, University of Florida, Gainesville, FL 32610-0277, USA; barteem@musc.edu (M.Y.B.); dhzheng@yahoo.com (D.Z.); 2 Centers for Personalized Diagnostics and Immunotherapy, Vaccines and Virotherapy, Biodesign Institute, Arizona State University, Tempe, AZ 85287-6401, USA; jyaron@asu.edu (J.R.Y.); liqiang.zhang@asu.edu (L.Z.); ihogue@asu.edu (I.B.H.); 3 Department of Surgery, BIDMC, Harvard Medical School, Boston, MA 02115, USA; liuliying2004@hotmail.com; 4 Department of Molecular Genetics and Microbiology, University of Florida, Gainesville, FL 32610, USA; wbullard@ufl.edu (W.L.B.); stibbe@ufl.edu (S.T.)

**Keywords:** MHV-68, gamma herpesvirus, Interleukin-10, macrophage, gastrointestinal, toxic megacolon

## Abstract

Inflammatory bowel disease (IBD) and *Clostridium difficile* infection cause gastrointestinal (GI) distension and, in severe cases, toxic megacolon with risk of perforation and death. Herpesviruses have been linked to severe GI dilatation. MHV-68 is a model for human gamma herpesvirus infection inducing GI dilatation in interleukin-10 (IL-10)-deficient mice but is benign in wildtype mice. MHV-68 also causes lethal vasculitis and pulmonary hemorrhage in interferon gamma receptor-deficient (IFNγR^−/−^) mice, but GI dilatation has not been reported. In prior work the Myxomavirus-derived anti-inflammatory serpin, Serp-1, improved survival, reducing vasculitis and pulmonary hemorrhage in MHV-68-infected IFNγR^−/−^ mice with significantly increased IL-10. IL-10 has been investigated as treatment for GI dilatation with variable efficacy. We report here that MHV-68 infection produces severe GI dilatation with inflammation and gut wall degradation in 28% of INFγR^-/-^ mice. Macrophage invasion and smooth muscle degradation were accompanied by decreased concentrations of T helper (Th2), B, monocyte, and dendritic cells. Plasma and spleen IL-10 were significantly reduced in mice with GI dilatation, while interleukin-1 beta (IL-1β), IL-6, tumor necrosis factor alpha (TNFα) and INFγ increased. Treatment of gamma herpesvirus-infected mice with exogenous IL-10 prevents severe GI inflammation and dilatation, suggesting benefit for herpesvirus-induced dilatation.

## 1. Introduction

Murine gammaherpesvirus-68 (MHV-68) is a naturally occurring rodent pathogen that is closely related to Epstein-Barr virus (EBV), Kaposi’s sarcoma–associated herpesvirus (KSHV), and Herpesvirus saimiri 2 (HVS-2) [1,2,3]. EBV and KSHV are associated with a range of diseases from infectious mononucleosis and lymphoma to nasopharyngeal carcinoma and Kaposi’s sarcoma. Herpesviruses such as EBV and the beta herpesvirus, cytomegalovirus (CMV), are also implicated in inflammatory bowel disease (IBD) [4,5,6,7,8,9]. MHV-68 infection causes a lethal sepsis in interferon gamma receptor-deficient (IFNγR^−/−^) mice with large vessel vasculitis, pulmonary hemorrhage, and consolidation, while in wildtype (WT) mice MHV-68 produces a benign infection that can become latent [1,2,3,10,11,12,13,14]. MHV-68 causes lymph node enlargement and splenomegaly and increases activated CD8 T cells in the blood [1,2,3,11,12,15,16,17]. Thus, although structurally the virus is more closely related to KSHV and HVS-2, MHV-68 pathogenesis in mice, in part, resembles that of EBV in humans. MHV-68 has thus become an accepted model for investigating numerous human diseases associated with systemic inflammation and particularly those with associations to herpesvirus infection.

Gastrointestinal (GI) dilatation, also referred to as GI distension, is a clinically significant condition with diverse etiologies including IBD, *Clostridium difficile* colitis after antibiotic treatment, irritable bowel syndrome, diabetes, functional dyspepsia, transient constipation, parasitic infection such as giardia or nematodes, bacterial food poisoning, celiac disease, severe peptic ulcer disease, bowel obstruction, immunosuppression and, in some cases, as a complication following abdominal surgery [4,5,6,7,8,9,18,19,20,21]. Patients with GI dilatation experience nausea, abdominal pressure, pain, or cramping. Importantly, in cases of IBD, *C. difficile* superinfection after antibiotic treatment, HIV infection in immunocompromised patients, or in patients with immunosuppression after transplant, severe GI distension can lead to a very serious dilatation termed toxic megacolon. Toxic megacolon is associated with extensive inflammation extending from the mucosal through to the smooth muscle layers and increased systemic cytokine release, with a risk of perforation and increased mortality [4,5,6,7,8,9]. MHV-68 infection is reported to cause GI dilatation and bacterial overgrowth in interleukin-10 (IL-10)-deficient mice [15], but GI dilatation in MHV-68 infection is not well understood and has not previously been reported in WT or IFNγR^−/−^ mouse models.

IL-10 has potent anti-inflammatory properties with a purported central role in limiting the host immune response to pathogens, thereby preventing damage induced by excess inflammation and maintaining normal tissue homeostasis. Deficient or aberrant IL-10 expression can enhance inflammatory responses to microbial challenge and is associated with the development of IBD and autoimmune diseases [15,21,22,23,24]. As noted, IL-10 has been closely linked to both GI dilatation [22,23] and MHV-68 infection [15] and IL-10-deficient (knock-out) mice display GI dilatation with spontaneous colitis [22,23]. Treatment with human IL-10 has been examined in clinical trials for IBD [15,21] with variable results and only modest benefits in Crohn’s disease at intermediate dosages used, but of interest, not at high doses. These findings may indicate that other factors from immune responses to the underlying causes for GI dilatation, such as superimposed viral infections, may alter response to IL-10 treatments in IBD. Newer formulations of IL-10 (e.g., DEKAVIL) that target fibronectin [25,26] and inflammatory foci are being tested in clinical trials. In one study, where higher IL-10 doses were administered, an increase was reported for inflammatory mediators such as tumor necrosis factor alpha (TNFα) and IFNγ [21,24]. While initially described as a T helper 2 (Th2) -derived cytokine, it should also be noted that IL-10 is not restricted to T cell subsets and is now known to be produced by a wide range of leukocytes including B cells, cytotoxic T cells, NK cells, mast cells, and granulocytes such as neutrophils and eosinophils [20,22,23,24].

In response to MHV-68 infection, IL-10 is produced by T cells, B cells and other non-T cell immunocytes [16,27]. In prior work, Nelson et al. [15] demonstrated marked increases in bacterial load and colon inflammation in IL-10-deficient mice. In contrast, Peacock et al. [27] reported that following intranasal infection with MHV-68, IL-10-deficient mice had reduced viral load but increased splenomegaly. In our prior work in MHV-68-infected IFNγR^−/−^ mice, treatment with a *ser*ine *p*rotease *in*hibitor (*serpin*) Serp-1, improved survival and was associated with a significant increase in IL-10 protein and gene expression. Serp-1 is an inhibitor of thrombotic and thrombolytic proteases (factor X, thrombin, tissue- and urokinase-type plasminogen activators, and plasmin) and can suppress associated inflammatory response activation [14]. In this prior study in MHV-68-infected IFNγR^−/−^ mice, Serp-1 treatment reduced vasculitis, inflammation, and pulmonary consolidation and hemorrhage with associated increases in IL-10, suggesting a potential beneficial effect for IL-10 in MHV-68 infection in IFNγR^−/−^ mice. [14].

In this study, we describe a previously unreported marked distension of the GI tract in MHV-68-infected IFNγR^−/−^ mice. MHV-68-infected mice with dilated GI sections had associated suppression of IL-10 expression and treatment with exogenous IL-10 significantly reduced GI inflammation and dilatation. This report establishes MHV-68 infection in IFNγR^−/−^ mice as a model for GI dilatation and supports a potential therapeutic role for IL-10 treatment in diseases associated with GI dilatation.

## 2. Materials and Methods

### 2.1. Ethics Statement

All animal studies conform to local and national guidelines for animal care and experimentation. The study protocol and experimental procedures were approved by the University of Florida and also Arizona State University Institutional Animal Care and Use Committees (IACUC) (UFL IACUC Protocol Approval #102004234, July 2013 and 2016, Title—Analysis of Viral Anti-Inflammatory Proteins as Anti-atherogenic Agents; ASU IACUC Protocol Approval #17-1549R, February 2017, Title—Treatment of Inflammatory Vasculitis and Transplant Rejection with Virus-derived Anti-Inflammatory Proteins and Peptides).

### 2.2. MHV-68 Virus Passage and Preparation

MHV-68 was prepared according to procedures described in previous publications [1,2,3,14]. MHV-68 viral stock solutions were produced in 3T12 cells (ATCC, Manassas, VA, USA). The culture medium for the 3T12 cells consisted of Dulbecco’s modified Eagle medium (DMEM), 10 mM HEPES, 2 mM L-Glutamine, 10% fetal calf serum as well as 1% penicillin/streptomycin. Infection of the cells at a multiplicity of infection of 0.1 occurred at 50% confluence. Cells underwent freeze thaw at seven days post-infection and the resulting lysate was transferred to polypropylene copolymer tubes (Nalgene Oak Ridge, Waltham, MA, USA) and centrifuged twice. Phosphate buffered saline was used to rinse the pellet. After being suspended again into medium and vortexed, the virus was stored at −80 °C in 250 µL aliquots. Virus titer was measured twice.

### 2.3. MHV-68 Infection in Interferon Gamma Receptor-Deficient (IFNγR^−/−^) Mice

IFNγR^−/−^ mice (129-*Ifngr1^tm1Agt^*/J; JAX #002702), 5–7 weeks of age were purchased from The Jackson Laboratory (Sacramento, CA, USA) and bred under specific pathogen free conditions. Littermate controls were used for all studies [14]. Each mouse was infected with a dose of 12.5 × 10^6^ PFU MHV-68 in DMEM administered via intraperitoneal (i.p.) injection as previously described [14]. Mice were treated with either saline control (100 µL, i.p. for 6 days) or IL-10 (200 ng/g in 100 μL, i.p. for 6 days, Biolegend #575806) (Table 1). Veterinary animal care staff and lab scientists monitored the mice daily to minimize potential suffering during the 3- to 15-day test period. Fifteen IFNγR^−/−^ mice were not infected, providing controls.

### 2.4. Histological and Morphometric Analysis

Mice were euthanized at follow up and organ tissue and blood samples harvested. All specimens from MHV-68-infected mice were cut into two equal lengths (colon 1.5 cm) and then cut into three parts, one each for histology, mRNA, and protein analysis, respectively. After fixing the samples in neutral buffered formalin (NBF), the samples were embedded in paraffin, and cut into 4 µm thick cross sections and stained with hematoxylin and eosin (H&E). The thickness of mucosa, submucosa, smooth muscle, and serosa were measured using an Olympus DP71 camera attached to a BX51 microscope (Olympus America Inc., Center Valley, PA, USA). Image Pro 6.0 software (MediaCybernetics Inc., Bethesda, MD, USA) was used for quantification. In our study, colon dilation was defined as a diameter more than 5.25 mm (more than 50% normal size) as measured at the widest diameter.

### 2.5. Western Blot

Tissue was lysed in denaturing lysis buffer (RIPA) adding dithiothreitol (DTT) to 20 mM before use, followed by SDS-PAGE and immunoblotting. The following antibodies were used for western blotting: rabbit monoclonal caldesmon antibody (Abcam ab45691, Cambridge, MA, USA), rabbit polyclonal IL-10 antibody (Abcam ab9735, Cambridge, MA, USA), and rabbit polyclonal β-actin antibody (Abcam ab52614, Cambridge, MA, USA).

### 2.6. Cytokine Assays

At the time of sacrifice, blood samples (0.8–1 mL) were obtained from MHV-68-infected mice, the blood was placed into chilled tubes containing ethylenediaminetetraacetic acid (EDTA), centrifuged, decanted, and the isolated plasma was then stored at −80 °C. To minimize inter-assay variability, plasma samples were measured simultaneously using a multiplex array for cytokines (Human MAP Base Kit LUH000, R&D Systems, Minneapolis, MN, USA). Internal controls for each cytokine were used to validate the multiplex array [14]. Plasma levels of interleukins IL-1β, IL-2, IL-4, IL-6, IL-8, tumor necrosis factor alpha (TNFα) and interferon gamma (INFγ) were measured using 20 μL undiluted samples. The absolute concentration of each cytokine was obtained by converting the relative fluorescence (Bio-Plex 200, BioRad Laboratories, Hercules, CA, USA) using calibration curves generated from known recombinant standards. Average sensitivities for cytokines were 0.3 pg/mL with a coefficient of variation ≤ 15%. The concentration of IL-10 in the plasma samples was also determined using an ELISA assay kit (Perkin Elmer Product number AL502CIF, Waltham, MA, USA).

### 2.7. Immunohistochemistry

For immunohistochemical staining, a rabbit specific horseradish peroxidase (HRP)/diaminobenzidine (DAB) detection IHC kit (ab64261, Abcam, Cambridge, MA, USA) was used following the manufacturer’s protocol, as previously described [14]. Tissue sections were stained with primary antibodies (rabbit monoclonal 1:100 for caldesmon ab45691, rabbit polyclonal 1:100 for CD3 ab93077, rabbit polyclonal 1:400 for CD11b ab75476; rabbit polyclonal 1:100 for CD83 ab64875; rabbit polyclonal 1:500 for CCR6 ab78429; rabbit pre and post anti-MHV-68 immune serum (Dr. H.W. Virgin, Washington University in St. Louis, St. Louis, MO, USA) [14], and rat monoclonal 1:100 for F4/80 ab15694, Abcam, Cambridge, MA, USA,). After primary antibody incubation, sections were then incubated with a secondary rabbit or rat specific HRP conjugate antibody, as indicated. DAB was then applied to the tissue and sections were counterstained with hematoxylin. Positively stained cells are brown.

### 2.8. Flow Cytometry

Splenocytes isolated from each mouse were stained with antibodies to surface or intracellular antigens and incubated for 30 min at room temperature. Labeled cells were washed and suspended in 150 µL of PBS and evaluated by flow cytometry, as previously described [14]. In brief, intracellular antigens were stained by incubating 500 µL suspensions of the cell pellets with fixation/permeabilization buffer (eBioscience, San Diego, CA, USA) in the dark for 45 min. Cells were then treated with permeabilization buffer (eBioscience, San Diego, CA, USA), and incubated with an intracellular antibody mix for an additional 30 min at 4 °C. All antibodies were obtained from eBiosciences and Biolegend (San Diego, CA, USA) (Supplemental Table S1). Supplemental Table S2 lists the immune cell types analyzed as well as corresponding fluorochrome-labeled antibodies used for analysis. Flow cytometry was performed with a CyAn ADP Analyzer (Dako, Ft Collins, CO, USA). Gatelogic software (eBioscience) was used for data analysis.

### 2.9. RT-PCR Analysis

MHV-68-infected mouse sample sections were collected in RNA*later* (Ambion, Austin, TX, USA). A RNeasy Mini kit was used to isolate RNA following the manufacturer’s protocol (QIAGEN, Valencia, CA, USA). Reverse transcription of the RNA to cDNA was performed using a Superscript VILO cDNA Synthesis kit (Invitrogen Corporation, Carlsbad, CA, USA). SYBR Green Core Reagent kit and 7300 RT-PCR system (Applied Biosystems, Austin, TX, USA) were used for real-time PCR. IL-10 (NM_010548.2; 105 bp amplicon); Primers used were forward sequence 5′-GCTCTTACTGACTGGCATGAG and reverse 5′-CGCAGCTCTAGGAGCATGTG. Other primers are listed in Supplemental Table S3.

### 2.10. Statistical Analysis

All data are presented as mean ± SD. Statistical analysis was performed by Analysis of Variance and by Student’s T test and Kruskal–Wallis test using SPSS 16.0 software (IBM, Armonk, New York, NY, USA) Statistical tests were corrected for multiple comparisons using the Benjamini-Hochberg method with a false discovery rate of 25% [28]. Rate comparison was analyzed by Pearson Chi-Square or Fisher’s Exact test. *p*-value < 0.05 was considered significant.

## 3. Results

### 3.1. GI Dilatation in Herpesvirus (MHV-68)-Infected IFNγR^−/−^ Mice

**Figure 1:**
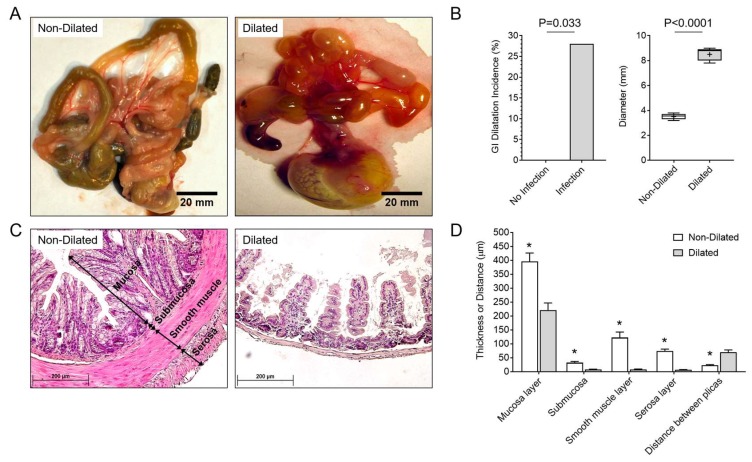
Pathological dilation of the GI tract was detected in MHV-68-infected IFNγR^−/−^ mice. (**A**) Non-dilated (left) GI section in uninfected mice (*n* = 15 IFNγR^-/-^ mice) and dilated (right) GI tract with transparent gut wall with minimal food and stool detected in the dilated gut of a saline-treated, infected mouse (*n* = 25); (**B**) Bar graph demonstrating 28% incidence of dilatation throughout the GI tract at 15 days after saline-treated MHV-68 infection and (right) mean colon diameters measured in MHV-68-infected IFNγR^−/−^ mice (*n* = 7 with dilatation; 8.5 ± 0.9 mm) were significantly increased in mice with dilated gut when compared to animals without dilatation (*n* = 18; 3.05 ± 0.35 mm, *p* < 0.0001); (**C**) GI tract histology demonstrates dilation of the colon sections from Herpesvirus (MHV-68)-infected IFNγR^−/−^ mice**,** Hematoxylin and Eosin (H&E) staining. (Left) Non-dilated colon cross section-mucosa, submucosa, smooth muscle, and serosa layers are labeled. (Right) Dilated colon with marked thinning of all layers. The layers of submucosa and the smooth muscle are almost completely absent in the dilated colon; (**D**) Thicknesses of layers of the mucosa, submucosa, smooth muscle and the serosal layers were significantly reduced in dilated gut sections (221.2 ± 25.56, 2.9 ± 1.1, 7.79 ± 1.9 and 6.68 ± 0.9 µm, respectively, *n* = 7) when compared to unaffected mice (395.9 ± 30.5, 32.5 ± 4.5, 122.7 ± 19.9 and 74.4 ± 6.8 µm, respectively, *n* = 18) (*p* < 0.001 for all analyses). Thinning of the gut layers are accompanied by markedly increased distance between plicas in dilated colon (70.1 ± 7.9 µm, *n* = 7), when compared to unaffected colon sections (23.3 ± 2.4 µm, *p* < 0.001, *n* = 18). * indicates a significance of *p* < 0.001.

Fifteen days after MHV-68 infection, 28% of infected IFNγR^−/−^ mice (*n* = 25, Table 1) exhibited excess dilatation throughout the whole GI tract, from the stomach to the colon. This gut dilatation was characterized by increased intestine diameter, mean 8.5 ± 0.9 mm, when compared to non-dilated, unaffected gut, mean 3.05 ± 0.35 mm, *p* = 0.009 (Figure 1A,B). In general, the dilated GI wall appeared transparent, indicating marked thinning of the wall (Figure 1A). Histologically, the mucosa, submucosa, smooth muscle, and serosa layers had significantly reduced thickness in dilated GI samples when compared to unaffected mice without GI dilatation (Figure 1C,D; *p* = 0.001). There was also an accompanying marked increase in the distance between the folds, plica, in the dilated GI samples, which is a strong indicator of GI dilatation. The layers of submucosa and smooth muscle were almost completely lost in dilated GI sections (Figure 1C,D).

### 3.2. Detection of MHV-68 and Macrophage Infiltrates the Colon in Infected IFNγR^−/−^ Mice

**Figure 2:**
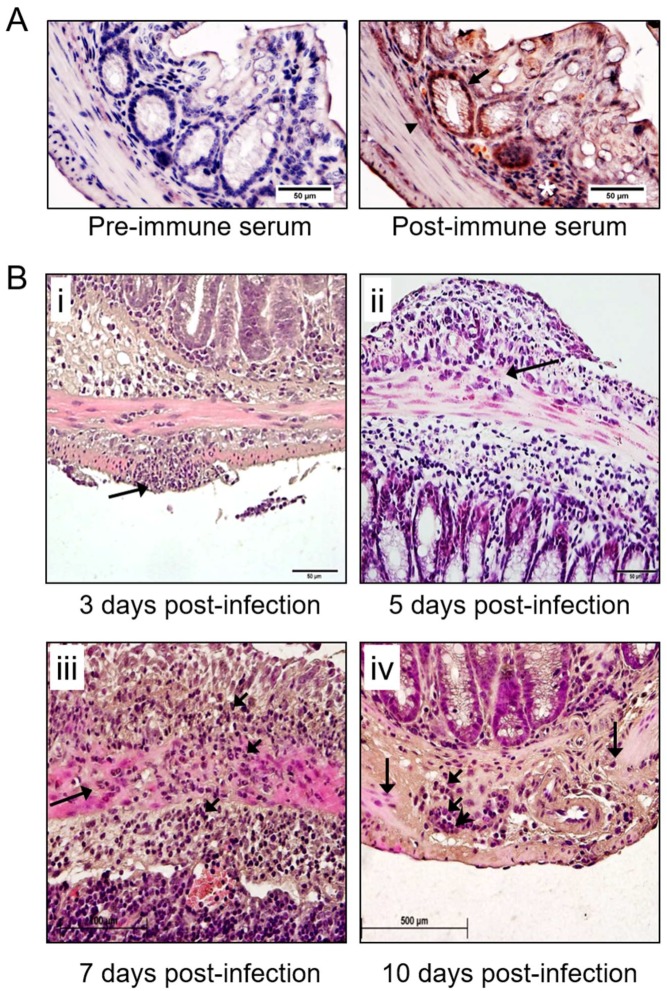
Sequential histological assessment of inflammatory cell infiltrates in the GI wall of MHV-68-infected IFNγR^−/−^ mice. MHV-68 virus staining was detected in infected mice at the base of colonic villi (arrow and arrowhead) and in invading mononuclear cells (asterisk). (**A**) Pre-immune control serum staining and post immune MHV-68 antibody staining of large intestine in MHV-68-infected mice. Histopathology (H&E staining) demonstrates mononuclear cell infiltrates at (**B**) i. 3 days, ii. 5 days, iii. 7 days and iv. 10 days post-infection. At 7 days a large infiltration of inflammatory cells is detected in the submucosa, serosa, and in particular in the smooth muscle layers. At this stage, the smooth muscle cells (SMC) layer is less clearly delineated and occupied by large numbers of inflammatory cells, indicating initial stages of breakdown of the SMC layer (*n* = 6 mice per follow up time). At 10 days after infection, SMC are markedly degraded and replaced by inflammatory cells with evidence for necrosis (*n* = 6). Short arrow points to the inflammatory cells, long arrow points to disrupted SMC layer.

To explore early changes in the GI tract after MHV-68 infection leading to colon dilatation, samples were harvested at 3, 5, 7 and 10 days post-infection (Figure 2; *n* = 6 mice for each follow up time point). Sequential histological analysis detected significant numbers of inflammatory cells trafficking into the submucosa, serosa, and in particular into the smooth muscle cells (SMC) layer. MHV-68 was detected by immunohistochemical staining in the colon after infection at sites of inflammation (Figure 2A,B). The SMC layer began to demonstrate breakdown at 7 days post-infection (Figure 2B). At 10 days post-infection the SMC layer was near completely degraded, with an associated extensive influx of inflammatory cells and necrosis (Figure 2B). Inflammatory cell invasion and SMC breakdown were thus much more pronounced at 7 and 10 days (Figure 2B), when compared to samples from 3 and 5 days follow up (Figure 2B).

**Figure 3:**
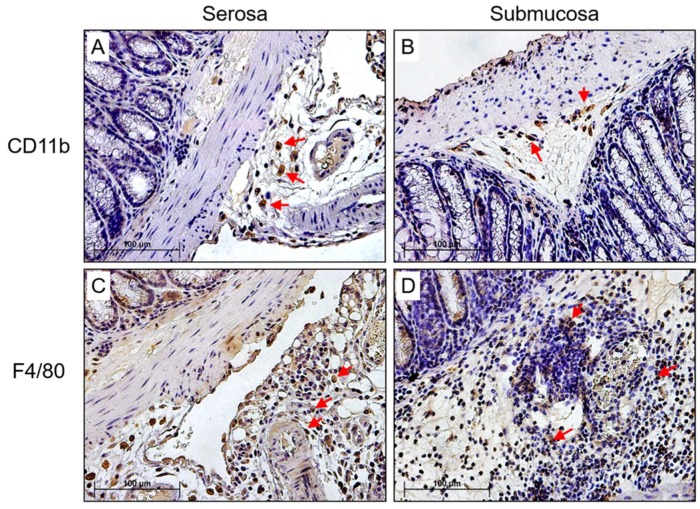
Macrophage and CD11b positive inflammatory mononuclear cell populations are predominant in the GI wall after MHV-68 infection in IFNγR^−/−^ mice and increase in the colon in areas of GI dilatation. CD11b staining detected positive cells in the (**A**) serosa and (**B**) submucosa respectively (*n* = 6). F4/80 positive staining was found at (**C**) serosal and (**D**) submucosal layers, respectively, at 7 days follow up. Red arrows indicate positively stained cells (*n* = 6) (magnification 400×).

To determine which specific inflammatory cells were associated with the degradation of the SMC layer, sections were stained in gut tissues isolated at 7 days follow up for monocyte, macrophage, and dendritic cell (DC) as well as T cell (TC) classes using antibody specific to CD11b (monocyte), F4/80 (macrophage), CD83 (DC), CD3 (TC), CD8 positive TC, and CCR6 (T memory), respectively (Figure 3). We found that CD11b positive monocytes and F4/80 positive macrophage stainings were predominant and increased in the SMC layer in dilated gut sections (Figure 3A,B, CD11b staining; Figure 3C,D, F4/80 staining). Minimal positive CD3, CD8, CCR6, and CD83 stained cells were detected.

### 3.3. Degradation of SMC Is Associated with GI Tract Dilatation in MHV-68 Infections

**Figure 4:**
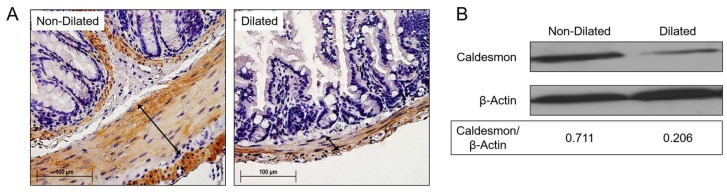
Degradation of smooth muscle cells in dilated colon in MHV-68-infected IFNγR^−/−^ mice. (**A**) Immunostaining for caldesmon, illustrated increased positive staining of submucosa and smooth muscle (arrows) in (left) non-dilated colon relative to (right) dilated colon. In dilated colon sections, only thinner smooth muscle layers were detected with weaker positive staining (*n* = 7). (**B**) Western blot demonstrates reduced expression of caldesmon protein in dilated GI samples when compared to unaffected colon samples, consistent with the histological immunostaining (*n* = 3).

To confirm that degradation and reduced numbers of SMCs were involved in the observed GI tract dilatation, colon sections were stained with antibody to caldesmon. Caldesmon is a calmodulin binding protein which tonically inhibits ATPase activity of myosin in SMCs [29]. Caldesmon serves as a mediating factor for Ca^2+^-dependent inhibition of SMC contraction and is used to trace the SMC content in the gut wall [29]. In dilated gut samples, immunohistochemical staining for caldesmon was significantly decreased when compared to unaffected or normal gut samples (Figure 4A). Reduced caldesmon content was confirmed by western blot (Figure 4B, β-actin loading control).

### 3.4. Enhanced Macrophage Invasion in Colon Samples is Accompanied by Decreased Systemic IL-10

**Figure 5:**
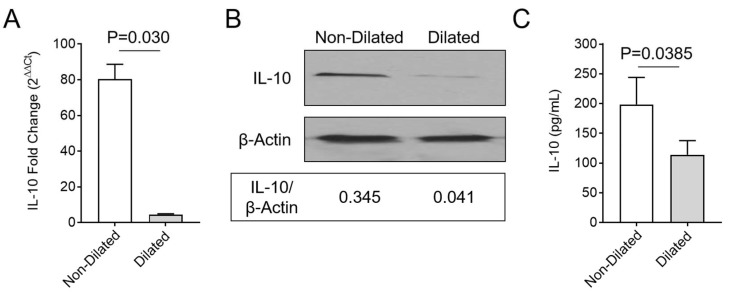
IL-10 expression after MHV-68 infection. IL-10 expression was significantly decreased at 15 days follow up in MHV-68-infected IFNγR^−/−^ mice with GI dilatation (*n* = 7). (**A**) The RNA level of IL-10 by real-time PCR in spleens of mice with GI dilatation was significantly decreased (*p* = 0.030) compared to infected mice without GI dilation (*n* = 18). (**B**) Western blot and densitometry analysis also demonstrated reduced IL-10 protein levels in spleen samples from infected mice with GI dilatation when compared to mice without GI dilatation (*n* = 3 each group). (**C**) Consistent with the results from the spleen assay, the plasma levels of IL-10 were also lower in mice with GI dilatation (*n* = 7) when compared to those with unaffected GI tracts (*n* = 18, *p* < 0.0385). Data presented as the mean ± SD.

To understand the underlying cause for monocyte and macrophage invasion into areas of colon dilation after MHV-68 infection, real-time quantitative PCR was performed. Gene expression for 21 genes with known associations to activation of inflammatory cell responses were analyzed at 15 days follow up. IL-10 gene expression was significantly reduced in spleens removed from mice with GI dilatation (Figure 5A; *p* = 0.030). Additionally, Factor II and Factor X were significantly upregulated (Supplemental Figure S1A,B; *p* < 0.05). Western blot analysis of the spleen, as well as ELISA for plasma protein, demonstrate reduced IL-10 protein levels in spleen and in circulating blood isolated from mice with GI dilatation, when compared to MHV-68-infected IFNγR^−/−^ mice without GI dilatation (Figure 5B,C; *p* = 0.0385).

### 3.5. GI Dilatation Is Associated with Decreased Th2 Cells, B Cells, Monocytes, and Dendritic Cells in Spleen Cell Isolates and Significantly Modified Plasma Cytokines

**Figure 6:**
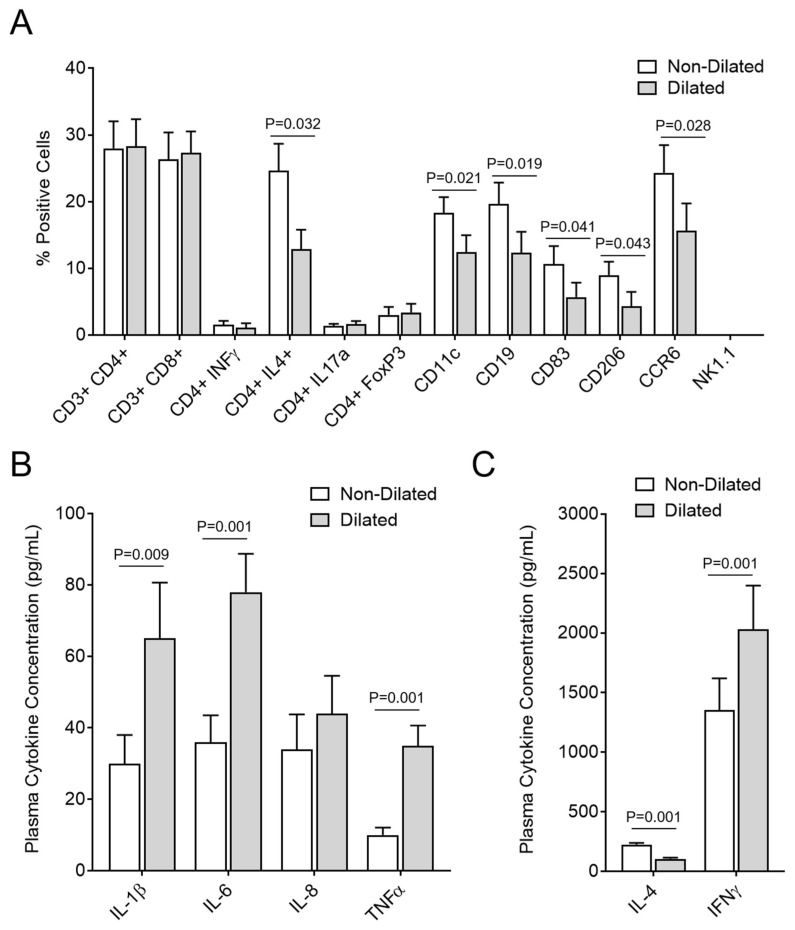
Inflammatory cells and cytokines are modified in MHV-68-infected IFNγR^−/−^ mice with GI dilation. (**A**) CD4^+^IL-4^+^ (Th2), CD11c (monocyte), CD19 (B cell), CD83 (mature dendritic cell), CD206 (immature dendritic cell), CCR6 (memory T cell) cells in mice with GI dilatation were significantly reduced compared to those in the mice without GI tract dilatation (*p* = 0.032, *p* = 0.021, *p* = 0.019, *p* = 0.041, *p* = 0.043, and *p* = 0.028, respectively). (**B**,**C**) Plasma levels of IL-1β, IL-6, TNFα, and INFγ increased in mice with dilated GI tract after MHV-68 infection, when compared to those without gut dilatation (*p* = 0.009, *p* = 0.001, *p* = 0.001 and *p* = 0.001, respectively). In contrast IL-4 decreased in mice with GI dilatation (*p* < 0.001). No significant difference in IL-8 was found between the two groups (*n* = 7 in mice with GI dilation, *n* = 18 mice without GI dilation; Data presented as mean ± SD). Cytokines in panel C are plotted with a differing Y axis than the cytokines in panel B due to the differing plasma levels of the detected cytokines.

To further investigate the cause for inflammatory cell reactions and GI tract dilatation, spleen cell isolates from mice with and without GI dilatation were assessed by flow cytometry (Figure 6). We detected significantly reduced CD4^+^IL-4^+^ (Th2), CD11c (monocyte), CD19 (B cell), CD83 (mature DC), CD206 (immature DC), CCR6 (memory TC) cells in mice with GI dilatation, when compared to mice without GI tract dilatation after MHV-68 infection (*p* < 0.032, *p* < 0.021, *p* < 0.019, *p* < 0.041, *p* < 0.043, and *p* < 0.028, respectively) (Figure 6A). Plasma cytokines IL-1β (*p* < 0.009), IL-6 (*p* < 0.001), TNF-α (*p* < 0.001) and IFNγ (*p* < 0.001) were significantly increased and IL-4 (*p* < 0.001) was significantly decreased in MHV-68-infected mice with GI dilatation (Figure 6B,C). Mice with GI dilatation thus had lower levels of detected monocytes and inflammatory cells in spleen cell isolates, in contrast to the mice without GI dilatation and the invading inflammatory cells seen after MHV-68 infection.

### 3.6. Treatment with IL-10 Significantly Reduces GI Inflammation and Dilatation in MHV-68-Infected IFNγR^−/−^ Mice

**Figure 7:**
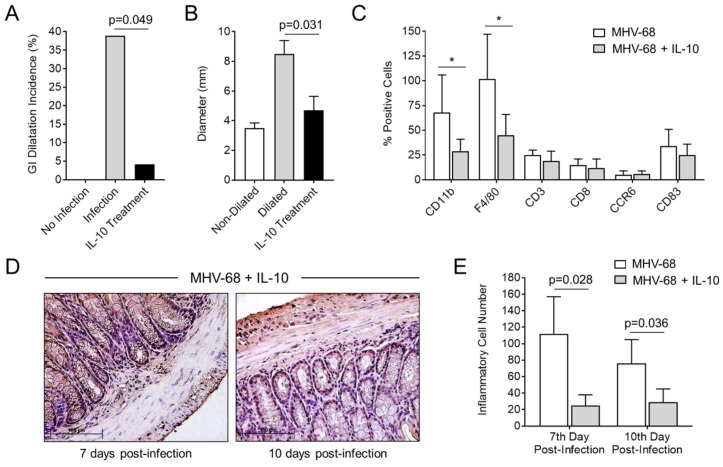
IL-10 treatment significantly reduces the incidence of GI Dilatation in MHV-68-infected IFNγR^−/−^ mice. (**A**) Both the incidence of MHV-68-induced GI dilatation (*p* = 0.049) and (**B**) colon diameter (*p* = 0.031) were reduced upon treatment with IL-10 (black bar) at 15 days follow up (*n* = 25 MHV-68-infected IFNγR^−/−^ mice with saline and *n* = 25 MHV-68-infected IFNγR^−/−^ mice with IL-10 treatment. (**C**) IL-10 treatment resulted in significant decreases in CD11b and F4/80 monocyte-macrophage counts in the colon of MHV-68-infected IFNγR^−/−^ mice. (**D**) Histological analysis demonstrated reduced invading inflammatory cells at 7 (left) and 10 days (right) post-infection, respectively (*n* = 6 mice treated with IL-10 per group at each time of follow up). (**E**) IL-10 treatment significantly reduces numbers of invading inflammatory cells in the GI wall at 7 and 10 days after infection (*p* = 0.028 and *p* = 0.036, respectively). * indicates *p* < 0.05.

To verify whether IL-10 treatment could reduce GI dilatation and inhibit the influx of monocyte/macrophage cells into the gut wall after MHV-68 infection, mice were treated with recombinant IL-10 (Figure 7). In this study, one out of 25 IL-10 treated mice developed mild GI dilatation (Figure 7A; *p* = 0.049). Compared to mice with severe GI dilatation (without IL-10 treatment), the diameter of the colon was significantly decreased by IL-10 (Figure 7B; *p* = 0.031). CD11b and F4/80 monocytes and macrophage cell infiltrates were significantly reduced with IL-10 treatments (Figure 7C; *p* < 0.05). Inflammatory cell numbers were also significantly reduced by IL-10 treatment at 7 and 10 days post-infection (Figure 7D,E; *p* = 0.028 day 7, *p* = 0.036 day 10).

## 4. Discussion

In the present study, we demonstrate marked dilatation of the entire GI tract after MHV-68 infection in IFNγR^−/−^ mice. Associated with the excess dilation there is concomitant loss of normal gut motility and marked macrophage invasion with associated loss of SMC. IL-10 expression is significantly reduced in mice with inflammation and dilatation in the colon and this observed GI distension is prevented by IL-10 treatment.

MHV-68 infection in IFNγR^−/−^ mice is generally considered a model for large vessel vasculitis and pulmonary hemorrhage with associated lung consolidation (pneumonia). The markedly dilated GI tract as reported here is a new observation for IFNγR^−/−^ mice with MHV-68 infection and may provide an additional model for gamma herpesvirus-induced bowel inflammation and toxic megacolon in IBD. MHV-68, a murine gamma herpesvirus, has been reported to cause GI tract dilatation in IL-10-deficient mouse models [15], but this has not been previously reported in the MHV-68-infected IFNγR^−/−^ mouse model.

The pathogenesis of GI distension is still poorly defined. A range of diverse etiologies has been linked to GI dilatation from impaired neuromotor function and electrolyte or metabolic imbalance to excess nitric oxide (NO) and cytokines that may reduce colon motor activity leading to dilatation in toxic megacolon [30,31,32,33,34,35,36,37]. Viral infections, specifically the herpesvirus, have also been linked to colonic dilatation. Herpes simplex virus infection is associated with GI tract neuronal dysfunction and a mouse model of toxic megacolon has been developed in mice with HSV infection of enteric neurons [36]. Epstein-Barr (EBV) and cytomegalovirus (CMV) are associated with severe GI dilatation in human cases of IBD [4,5,6,7,8,9]. CMV has also been associated with C. difficile infections after antibiotic treatment and in immunosuppressed patients [4,5,6,7]. Treatment with antiviral agents in severe GI dilatation has been suggested with some reported success in severe disease [4,5].

In this study, MHV-68 was inoculated intraperitoneally (i.p.), thus one of the first and the most readily accessed organs is the gut and/or other abdominal organs. Our results may in part reflect the use of an i.p. infection route as opposed to a nasal inoculum. The immediate exposure of the virus to the gut may also explain why macrophage and monocytes activate early in response to MHV-68 located in the GI tract at the onset of infection. Macrophages are highly specialized in the removal of dying or dead cells and cellular debris. In Figure 3 we demonstrate markedly increased monocytes in the SMC gut layer at 7 days after infection, as well as positive staining for MHV-68 in the gut at sites of inflammatory cell invasion. At this time, the integrity of the SMC layer was still maintained. At 10 days after infection, discontinuity of the SMC layer was observed with associated phagocytic macrophage infiltrates causing muscle layer inflammation and degradation of the contents of injured muscle fibers. This breakdown of smooth muscle cell layers by macrophage lysis is predicted to be followed by reduced smooth muscle motility and GI dilation.

After infection in IFNγR^−/−^ mice, MHV-68 may traffic to the spleen through the circulating blood, resulting in altered leukocyte response and leading to the observed decrease in Th2, B cells, monocyte, and dendritic cells, which was accompanied by decreased IL-10. This series of events may contribute to the invasion of inflammatory cells into sites of infection in the GI tract, increasing macrophage activation and SMC breakdown in the gut. MHV-68 in the process of direct and random movement to the GI wall may be phagocytosed by macrophages, after activation and the release of pro-inflammatory cytokines, such as IL-1β, IL-6, TNFα and INFγ. These cytokines may recruit more inflammatory cells, producing proteinases that break down the smooth muscle cell layer and cause GI tract dilatation. In our study, IL-10 levels decreased in IFNγR^−/−^ mice with GI tract dilatation after infection, potentially leading to decompensation and exacerbating inflammatory reactions. Prior work with the MHV-68 M2 protein, a unique viral protein, demonstrated that MHV-68 modifies B cell signaling and induces cellular IL-10 secretion [36], potentially making B cells more responsive to IL-10 signaling and leading to proliferation and enhanced survival of M2-expressing primary B cells in culture. In our study, increased macrophage and monocyte cells are observed in the wall of dilated GI tract sections when compared to the unaffected mice. We demonstrate here that the accumulation of macrophage and monocyte at the GI wall is associated with increased inflammatory cytokines IL-1β, IL-6, TNFα and INFγ-cytokines which signal recruitment of more inflammatory cells to sites of infection.

The response to IL-10 treatment in this mouse model supports treatment of gamma herpesvirus-associated toxic GI dilatation via treatments that increase IL-10 activity. IL-10 plays a critical role in regulating homeostasis, resolving inflammation during acute infection or tissue injury, at both local and systemic levels. Dysregulation of IL-10 may lead to more severe immunopathology through enhanced or sustained inflammatory response. In this study, GI tract dilatation is closely associated with marked macrophage invasion, breakdown of smooth muscle cell layers and correlates with reduced IL-10 levels. Colon dilation and concomitant inflammation was reduced with IL-10 treatment given at the onset of MHV-68 infection. Decreased levels of IL-10 were associated with decreased Th2, B cells, monocyte and DCs in isolated spleen cells. Prior studies have verified that MHV-68 infection leads to increased IL-10 as a compensatory mechanism to control inflammation, with enhanced murine B cell viability and human B cell proliferation, suppressing Th2 responses through modulation of macrophage function as well as inhibition of cytokine production. While IL-10, or new formulations of IL-10, have not yet been reported to demonstrate clear benefit in IBD, investigations are in progress [18,20,24]. In studies in Crohn’s disease with human IL-10 treatment, higher dose IL-10 treatment led to elevated levels of inflammatory mediators, specifically IFNγ and TNFα. In the model used here for these studies, MHV-68 infection led to severe disease in IFNγR-deficient mice. IL-10 treatment may thus have greater efficacy in the model used here as the mice lack the IFNγ receptor. IL-10 treatment may also have improved efficacy when given together with agents that have the capacity to reduce IFNγ activity, but this will require further investigation.

## 5. Conclusions

In summary, MHV-68 infection in IFNγR^−/−^ mice causes severe GI dilatation with attendant macrophage invasion and SMC degradation that is closely associated with reduced IL-10 expression. Treatment with IL-10 significantly reduces GI dilation as well as inflammatory cell invasion in the gut wall of MHV-68-infected IFNγR^−/−^ mice. IL-10 may be beneficial in treatment for GI tract inflammation and dilatation in IBD-associated with gamma herpes viral infections, potentially with greater efficacy when coupled with reduced IFNγ responses.

## Figures and Tables

**Table 1:** Numbers of mice with and without MHV-68 infection.

	Number IFNγR^−/−^ Mice No MHV-68 Infection	Time to Follow Up–Number of MHV-68 Infected IFNγR^−/−^ Mice				
Treatment	15 days	3 days	5 days	7 days	10 days	15 days
Saline	15	6	6	6	6	25
IL-10	0	6	6	6	6	25
Total numbers MHV-68 infected mice	0	12	12	12	12	50
Total number mice ± MHV-68 infection	Total—15 mice− MHV-68 infection	Total—98 mice + MHV-68 infection				

## Supplementary Materials

The following are available online at http://www.mdpi.com/1999-4915/10/10/518/s1, Table S1: Antibodies used in cell staining. Table S2: Immune cell types and corresponding fluorochrome-labeled antibodies. Table S3: Primer sequence used in quantitative RT-PCR assays. Figure S1. Upregulation of blood clotting factors in the spleen associated with MHV-68-induced colon dilation.

## References

[1] Tibbetts S.A., Loh J., van Berkel V., McClellan J.S., Jacoby M.A., Kapadia S.B., Speck S.H., Virgin H.W. (2003). Establishment and Maintenance of Gammaherpesvirus Latency Are Independent of Infective Dose and Route of Infection. J. Virol..

[2] Tripp R.A., Hamilton-Easton A.M., Cardin R.D., Nguyen P., Behm F.G., Woodland D.L., Doherty P.C., Blackman M.A. (1997). Pathogenesis of an infectious mononucleosis-like disease induced by a murine gamma-herpesvirus: Role for a viral superantigen?. J. Exp. Med..

[3] Canny S.P., Goel G., Reese T.A., Zhang X., Xavier R., Virgin H.W. (2014). Latent Gammaherpesvirus 68 Infection Induces Distinct Transcriptional Changes in Different Organs. J. Virol..

[4] Ciccocioppo R., Racca F., Paolucci S., Campanini G., Pozzi L., Betti E., Riboni R., Vanoli A., Baldanti F., Corazza G.R. (2015). Human cytomegalovirus and Epstein-Barr virus infection in inflammatory bowel disease: Need for mucosal viral load measurement. World J. Gastroenterol..

[5] Pillet S., Pozzetto B., Roblin X. (2016). Cytomegalovirus and ulcerative colitis: Place of antiviral therapy. World J. Gastroenterol..

[6] Chan K.S., Lee W.Y., Yu W.L. (2016). Coexisting cytomegalovirus infection in immunocompetent patients with Clostridium difficile colitis. J. Microbiol. Immunol. Infect..

[7] Autenrieth D.M., Baumgart D.C. (2012). Toxic megacolon. Inflamm. Bowel Dis..

[8] Roth J.L., Valdes-Dapena A., Stein G.N., Bockus H.L. (1959). Toxic megacolon in ulcerative colitis. Gastroenterology.

[9] Caprilli R., Vernia P., Colaneri O., Frieri G. (1980). Risk factors in toxic megacolon. Dig. Dis. Sci..

[10] Virgin H.W., Latreille P., Wamsley P., Hallsworth K., Weck K.E., Dal Canto A.J., Speck S.H. (1997). Complete sequence and genomic analysis of murine gammaherpesvirus 68. J. Virol..

[11] Sunil-Chandra N.P., Arno J., Fazakerley J., Nash A.A. (1994). Lymphoproliferative disease in mice infected with murine gammaherpesvirus 68. Am. J. Pathol..

[12] Weck K.E., Dal Canto A.J., Gould J.D., O’Guin A.K., Roth K.A., Saffitz J.E., Speck S.H., Virgin H.W. (1997). Murine gamma-herpesvirus 68 causes severe large-vessel arteritis in mice lacking interferon-gamma responsiveness: A new model for virus-induced vascular disease. Nat. Med..

[13] Dal Canto A.J., Swanson P.E., O’Guin A.K., Speck S.H., Virgin H.W. (2001). IFN-γ action in the media of the great elastic arteries, a novel immunoprivileged site. J. Clin. Investig..

[14] Chen H., Zheng D., Abbott J., Liu L., Bartee M.Y., Long M., Davids J., Williams J., Feldmann H., Strong J. (2013). Myxomavirus-derived serpin prolongs survival and reduces inflammation and hemorrhage in an unrelated lethal mouse viral infection. Antimicrob. Agents Chemother..

[15] Nelson D.A., Petty C.C., Bost K.L. (2009). Infection with murine gammaherpesvirus 68 exacerbates inflammatory bowel disease in IL-10-deficient mice. Inflamm. Res..

[16] Stevenson P.G., Efstathiou S. (2005). Immune Mechanisms in Murine Gammaherpesvirus-68 Infection. Viral Immunol..

[17] Tarakanova V.L., Suarez F., Tibbetts S.A., Jacoby M.A., Weck K.E., Hess J.L., Speck S.H., Virgin H.W. (2005). Murine Gammaherpesvirus 68 Infection Is Associated with Lymphoproliferative Disease and Lymphoma in BALB 2 Microglobulin-Deficient Mice. J. Virol..

[18] Fekety R., Shah A.B. (1993). Diagnosis and treatment of Clostridium difficile colitis. JAMA.

[19] O’Garra A., Barrat F.J., Castro A.G., Vicari A., Hawrylowicz C. (2008). Strategies for use of IL-10 or its antagonists in human disease. Immunol. Rev..

[20] Wang X., Wong K., Ouyang W., Rutz S. (2017). Targeting IL-10 Family Cytokines for the Treatment of Human Diseases. Cold Spring Harb. Perspect. Biol..

[21] Katsanos K.H., Papadakis K.A. (2017). Inflammatory bowel disease: Updates on molecular targets for biologics. Gut Liver.

[22] Kühn R., Löhler J., Rennick D., Rajewsky K., Müller W. (1993). Interleukin-10-deficient mice develop chronic enterocolitis. Cell.

[23] Sellon R.K., Tonkonogy S., Schultz M., Dieleman L.A., Grenther W., Balish E., Rennick D.M., Sartor R.B. (1998). Resident enteric bacteria are necessary for development of spontaneous colitis and immune system activation in interleukin-10-deficient mice. Infect. Immun..

[24] Ouyang W., Rutz S., Crellin N.K., Valdez P.A., Hymowitz S.G. (2011). Regulation and Functions of the IL-10 Family of Cytokines in Inflammation and Disease. Annu. Rev. Immunol..

[25] Schwager K., Kaspar M., Bootz F., Marcolongo R., Paresce E., Neri D., Trachsel E. (2009). Preclinical characterization of DEKAVIL (F8-IL10), a novel clinical-stage immunocytokine which inhibits the progression of collagen-induced arthritis. Arthritis Res. Ther..

[26] Galeazzi M., Baldi C., Schwager K., Neri D., Giovannoni L., Selvi E. (2013). A phase IB clinical trial with dekavil (F8-IL10), an anti-inflammatory immunocytokine for the treatment of rheumatoid arthritis, used in combination with methotrexate. Ann. Rheum. Dis..

[27] Peacock J.W., Bost K.L. (2001). Murine gammaherpesvirus-68-induced interleukin-10 increases viral burden, but limits virus-induced splenomegaly and leukocytosis. Immunology.

[28] Benjamini Y., Hochberg Y. (1995). Controlling the false discovery rate: A practical and powerful approach to multiple testing. J. R. Stat. Soc..

[29] Pritchard K., Moody C.J. (1986). Caldesmon: A calmodulin-binding actin-regulatory protein. Cell Calcium.

[30] Storsteen K.A., Kernohan J.W., Bargen J.A. (1953). The myenteric plexus in chronic ulcerative colitis. Surg. Gynecol. Obstet..

[31] Collins S.M., Hurst S.M., Main C., Stanley E., Khan I., Blennerhassett P., Swain M. (1992). Effect of inflammation of enteric nerves. Cytokine-induced changes in neurotransmitter content and release. Ann. N. Y. Acad. Sci..

[32] Geboes K., Collins S. (1998). Structural abnormalities of the nervous system in Crohn’s disease and ulcerative colitis. Neurogastroenterol. Motil..

[33] Reddy S.N., Bazzocchi G., Chan S., Akashi K., Villanueva-Meyer J., Yanni G., Mena I., Snape W.J. (1991). Colonic motility and transit in health and ulcerative colitis. Gastroenterology.

[34] Latella G., Papi C. (2012). Crucial steps in the natural history of inflammatory bowel disease. World J. Gastroenterol..

[35] Latella G., Vernia P., Viscido A., Frieri G., Cadau G., Cocco A., Cossu A., Tomei E., Caprilli R. (2002). GI distension in severe ulcerative colitis. Am. J. Gastroenterol..

[36] Khoury-Hanold W., Yordy B., Kong P., Kong Y., Ge W., Szigeti-Buck K., Ralevski A., Horvath T.L., Iwasaki A. (2016). Viral Spread to Enteric Neurons Links Genital HSV-1 Infection to Toxic Megacolon and Lethality. Cell Host Microbe.

[37] Rangaswamy U.S., Speck S.H. (2014). Murine Gammaherpesvirus M2 Protein Induction of IRF4 via the NFAT Pathway Leads to IL-10 Expression in B Cells. PLoS Pathog..

