# Supplementary material for: Mouse Gamma Herpesvirus MHV-68 Induces Severe Gastrointestinal (GI) Dilatation in Interferon Gamma Receptor-Deficient Mice (IFNγR−/−) That Is Blocked by Interleukin-10
Source: Viruses. 2018 Sep 23;10(10):518. doi: 10.3390/v10100518 (PMC6213885; doi:10.3390/v10100518)
Supplement: Supplementary file 1 [file viruses-10-00518-s001.pdf]

Supplemental Material

Supplemental Table S1. Antibodies utilized in cell staining.

Name	Company	Catalog	Usage	Concentration
Rabbit monoclonal caldesmon	Abcam	Ab32330	Immunohistochemistry	1:100
Rabbit polyclonal to CD3	Abcam	ab93077	Immunohistochemistry	1:100
Rabbit polyclonal to CD11b	Abcam	ab75476	Immunohistochemistry	1:400
Rabbit polyclonal to CCR6	Abcam	ab78429	Immunohistochemistry	1:500
Rabbit polyclonal to CD83	Abcam	ab64875	Immunohistochemistry	1:100
Rat monoclonal F4/80	Abcam	ab15694	Immunohistochemistry	1:100
Anti-CD3-PerCP-Cy5.5	eBioscience	45-0031-80	Flow cytometry	1:100
Anti-CD8-APC-eFluor780	eBioscience	47-0081-80	Flow cytometry	1:100
Anti-CD4-PE-Cy7	eBioscience	25-0041-81	Flow cytometry	1:100
Anti-IFNγ-FITC	eBioscience	11-7311-71	Flow cytometry	1:100
Anti-IL4-PE	eBioscience	12-7041-71	Flow cytometry	1:100
Anti-IL17a-AF647	eBioscience	51-7177-80	Flow cytometry	1:100
Anti-FoxP3-eFluor450	eBioscience	48-5773-80	Flow cytometry	1:100
Anti-CD19-Cy7	eBioscience	25-0193-81	Flow cytometry	1:100
Anti-CD34-PerCP-Cy5.5	Biolegend	128607	Flow cytometry	1:100
Anti-NK1.1-eFluor450	eBioscience	48-5941-80	Flow cytometry	1:100
Anti-CD11b-APC-eFluor780	eBioscience	47-0112-80	Flow cytometry	1:100
Anti-CD11c-APC-eFluor780	eBioscience	47-0114-80	Flow cytometry	1:100
Anti-CD83-PE	Biolegend	121507	Flow cytometry	1:100
Anti-CD206-FITC	Biolegend	123005	Flow cytometry	1:100
Anti-CCR6-APC	Biolegend	129813	Flow cytometry	1:100

Supplemental Table S2. Immune cell types and corresponding fluorochrome-labeled antibodies.

Cell Types	Marker
Cytotoxic T cell	Anti-CD3-PerCP-Cy5.5; Anti-CD8-APC-eFluor780
T helper cell	Anti-CD3-PerCP-Cy5.5; Anti-CD4-PE-Cy7
Th1 cell	Anti-IFNγ-FITC
Th2 cell	Anti-IL4-PE
Th17 cell	Anti-IL17a-AF647
Treg cell	Anti-FoxP3-eFluor450
B cell	Anti-CD19-Cy7
Hematopoietic stem cell	Anti-CD34-PerCP-Cy5.5
NK cell	Anti-NK1.1-eFluor450
Monocyte	Anti-CD11b/c-APC-eFluor780
Dendritic cell (Mature)	Anti-CD83-PE
Dendritic cell (Immature)	Anti-CD206-FITC
Memory T cell	Anti-CCR6-APC

Supplemental Table S3. Primer sequence utilized in quantitative RT-PCR assays.

Gene Name	Forward Primer	Reverse Primer
Factor II	CCGAAAGGGCAACCTAGAGC	GGCCCAGAACACGTCTGTG
Factor X	GAGGGACACCTACGACTATG	GCCCAGTCTTTCTGAGGCA
uPAR	CAGAGCTTTCCACCGAATGG	GTCCCCGGCAGTTGATGAG
tPA	AACGCAGACAACTTACCAACA	GTTCGCTGCAACTTCGGA
PAI1	TTCAGCCCTTGCTTGCCTC	ACACTTTTACTCCGAAGTCGGT
PAR2	TCCGGTCGTCTACATTATTGTGT	AGGGGGAACCAGATGACAGAG
IL17	TCAGCGTGTCCAAACACTGAG	GACTTTGAGGTTGACCTTCACAT
L-selectin	TCTGGGAAATGGAACGATGACG	CCGTAATACCCTGCATCACAGAT
CCL5	ACCAGTGGCAAGTGCTCCA	GCACACACTTGGCGGTTCTT
Traf3	CAGCCTAACCCACCCCTAAAG	TCTTCCACCGTCTTCACAAAC
TNFa	CCCTCACACTCAGATCATCTTCT	GCTACGACGTGGGCTACAG
CCR1	AGCCAGTACGAAGTGATCTGC	CTGCGAGCCCAGTGACAAA
IFNγ	ATGAACGCTACACACTGCATC	CCATCCTTTTGCCAGTTCCTC
CD3	GTGGAACACTTTCTGGGGCATCCTG	TGTTCTCGGCATCGTCCTGGCA
CD4	ACCATGTGCCGAGCCATCTCTCTT	CCAGCACCAGCGTCTTCCCTTG
FoxP3	CCCATCCCCAGGAGTCTTG	ACCATGACTAGGGGCACTGTA
Addressin	CTGAGCCCTACATCCTGACCT	GCTTCACAGAGTAGCTCCCAG
bFGF	GAGTTGTGTCTATCAAGGGAGTG	CCGTCCATCTTCCTTCATAGC
Ms-Tbet-F	AACCGCTTATATGTCCACCCA	CTTGTTGTTGGTGAGCTTTAGC
OPG	CAGCATCGCTCTGTTCCTGTA	CTGCGTTTTCATGGAGTCTCA
GAPDH	AGGTCGGTGTGAACGGATTTG	TGTAGACCATGTAGTTGAGGTCA

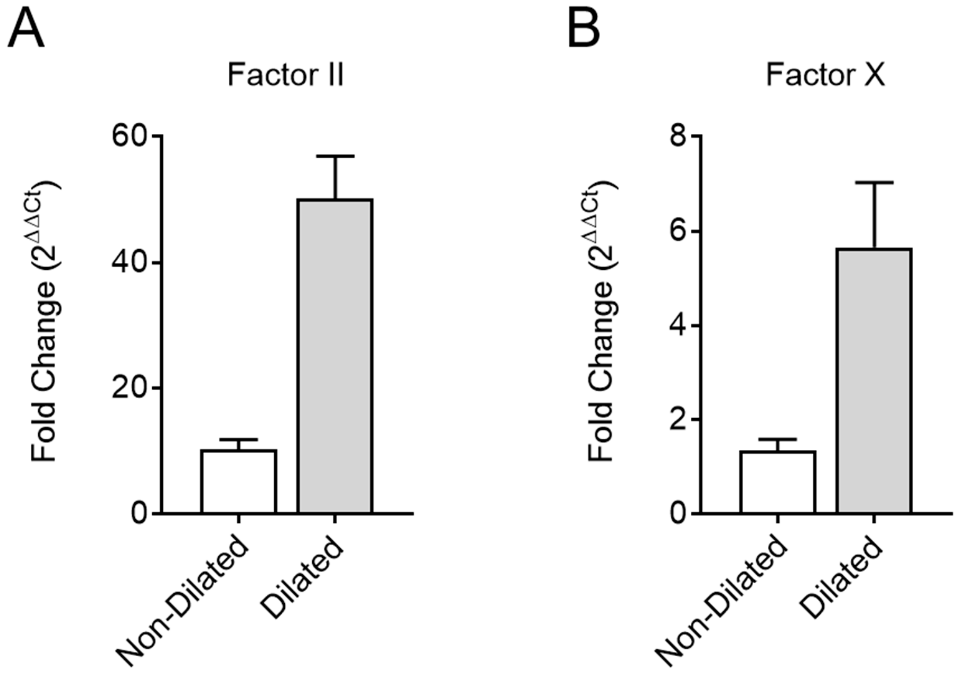

Supplemental Figure 1. Upregulation of blood clotting factors in the spleen associated with MHV-68-induced colon dilation. Factor II (A) and Factor X (B) gene expression was differentially regulated between saline-treated MHV-68-infected mice exhibiting non-dilated (white bars) or dilated (gray bars) colons. Results $P < 0.05$ are considered significant.
